# Planetary health for health systems: A scoping review and content analysis of frameworks

**DOI:** 10.1371/journal.pgph.0004710

**Published:** 2025-06-17

**Authors:** Nicole Redvers, Kyla Wright, Jamie Hartmann-Boyce, Sarah Tonkin-Crine

**Affiliations:** 1 Schulich School of Medicine and Dentistry, University of Western Ontario, London, Ontario, Canada; 2 Department for Continuing Education, University of Oxford, Oxford, United Kingdom; 3 Nuffield Department of Primary Care Health Sciences, University of Oxford, Oxford, United Kingdom; 4 School of Public Health Sciences, University of Waterloo, Waterloo, Ontario, Canada; 5 Department of Health Policy and Promotion, University of Massachusetts Amherst, Amherst, Massachusetts, United States of America; Griffith University, AUSTRALIA

## Abstract

Planetary health movements have advanced substantially within the last ten years with new frameworks and models being considered within health systems in varied contexts. Despite advancements, there continues to be an overall lack of accessible and regional- or field-specific planetary health frameworks to inform health systems. We therefore set out to conduct a scoping review to identify current planetary health-related frameworks that have been developed for health systems. We systematically searched the following electronic databases up to November 2023: Medline, CAB Abstracts, and Scopus; and carried out manual searches in Overton, Policy Commons, Google, and Google Scholar. We engaged a two-stage article review process, then used content analysis to identify the different domains. We identified six overarching categories within the planetary health-related frameworks including: 1) health system and environmental impacts; 2) vision, advocacy, leadership, and communication elements; 3) key structural components for environmentally sustainable health systems; 4) climate resiliency and environmental sustainability of healthcare facilities and systems; 5) climate-resilient and sustainable technologies and infrastructure; and 6) evaluation and accountability mechanisms. Regional, national, and international governments, funding agencies, and organizations are called to support greater research and implementation work around planetary health-informed health systems change while considering existing frameworks. Better inclusion of all facets of planetary health (e.g., biodiversity), as well as key acknowledgement and work with other knowledge systems (e.g., Indigenous Peoples and their knowledge systems) are needed to ensure planetary health-related frameworks are grounded in what we are trying to protect—the planet itself.

## Introduction

There have been increasing calls to center climate mitigation and adaptation planning, preparedness, and action within health systems globally. With a greater appreciation for the co-occurring global crises, planetary health approaches within healthcare spaces that are inclusive of climate change, biodiversity loss, and pollution are increasingly being looked to. This greater appreciation was highlighted during the first ever Health Day held at the 2023 Conference of the Parties (COP) summit (i.e., United Nations Climate Change Conference) where links between health and climate change were platformed [1]. The Health Day resulted in a new Declaration on Climate and Health, signed by 124 countries, with the intent “to place health at the heart of climate action and accelerate the development of climate-resilient, sustainable and equitable health systems” [2]. For the very first time at a COP UN Climate Conference, Health Ministers also attended, which signaled a key shift in “how climate policies are considered” [2].

Broader planetary health movements have advanced substantially within the last ten years with new frameworks and models being considered and developed within health systems in varied contexts, including within health professions education [3]. To this end, a task force was convened between 2019 and 2021 to develop ‘The Planetary Health Education Framework’ which considers five foundational domains believed to be “the essence of planetary health knowledge, values, and practice” [4] (i.e., interconnection within nature, the Anthropocene and health, systems thinking and complexity, equity and justice, and movement building and systems change [5]). Despite key movements within planetary health education, there continues, however, to be an overall lack of accessible, and regional- or field-specific planetary health-related frameworks to inform health systems in their goals towards climate-resilient and low-carbon health system. The World Health Organization (WHO) has published key high level operational frameworks [6,7]; however, they are at the broad health systems level, and it is not clear whether other frameworks exist that either advance, or are built from the WHO-related operational frameworks already developed. Given this, identifying similarities and differences across existing frameworks could support organizations in the development of their own planetary health-related frameworks.

Frameworks or models and their respective domains have often informed change processes within healthcare contexts [8]. Frameworks also often guide the principles in health-system change, including in quality improvement (QI) (e.g., Institute of Medicine’s (IOM) domains of healthcare quality) [9]. With this, well-built planetary health frameworks within health systems may facilitate easier routes towards the implementation of key climate adaptation and mitigation measures; to ensure both a reduction in health systems’ impacts on the environment, as well as climate change-related impacts on health systems. Although frameworks for planetary health currently exist, they vary in scope and content, and people seeking to propose and enact system changes to support planetary health may not be clear on where to find these frameworks, and which elements apply to their work. We therefore set out to conduct a scoping review to identify current planetary health-related frameworks that have been developed for health systems. More specifically, our review sought to: 1) Identify planetary health-related frameworks proposed, developed, or currently used within health systems; 2) describe the content of these frameworks, their stage of implementation, and any accountability mechanisms noted; and 3) to identify the framework elements commonly considered as critical to the development and functioning of environmentally sustainable healthcare systems. Our long-term goal outside of this current scoping review, is through a series of studies, to inform the creation of community-informed frameworks that are responsive to health systems, communities, and the planet.

## Methods

Our scoping review followed the methodology developed by Arksey and O’Malley (2005) [10], that was further refined by Peters et al. (2021) [11]. The PRISMA-ScR extension [12] was used to ensure appropriate reporting standards for scoping reviews. We pre-registered our protocol on the Open Science Framework (OSF) [13]. Our review question was: What planetary health-related frameworks are available to guide the application and/or evaluation of planetary health approaches within health systems?

### Information sources and search strategy

A medical research librarian at the University of Oxford was consulted to co-create a systematic search strategy. Example search terms are outlined in Table 1. The following electronic databases were searched as a part of the review to identify relevant articles: Medline, CAB Abstracts, and Scopus. The first 100 articles were reviewed for relevance in both Google and Google Scholar using adapted search terms for Google [e.g., (“planetary health” OR “planetary healthcare”) framework; (“green health” OR “green healthcare”) framework]. We also carried out manual searches in Overton and Policy Commons. The reference lists of all articles that met the inclusion criteria were searched to further identify relevant articles not found during the initial search steps.

**Table 1:** Example search terms for Scopus.

Database	Search Terms
Scopus	(TITLE (framework* OR tool* OR model* OR plan*) OR TITLE (implement*) OR ABS (implement* W/10 (framework* OR tool* OR model* OR plan*)) OR ABS (project* W/3 (framework* OR tool* OR model* OR plan*))) AND (TITLE ({planetary health*}) OR TITLE (green* W/1 (healthcare OR “health care” OR “health system*”)) OR (TITLE-ABS-KEY ((climat* W/1 change) OR “carbon footprint”) OR TITLE-ABS-KEY ((climat* OR environment*) W/3 (resilien* OR sustainab*)) OR TITLE-ABS-KEY (((low* OR reduc* OR decreas* OR neutral*) W/2 carbon) OR (decarbon* OR de-carbon*)) AND TITLE (healthcare OR “health care” OR “health* system*” OR “health* service*”) OR ABS (sustainab* W/3 (healthcare OR “health care” OR “health* system*” OR “health* service*”)) OR ABS (deliver* W/3 (healthcare OR “health care” OR “health* system*” OR “health* service*”)) OR ABS (develop* W/3 (healthcare OR “health care” OR “health* system*” OR “health* service*”))))

### Eligibility and study selection

All articles identified through the search strategy were transferred into Covidence review software (v2721 a9510157) to facilitate the article selection process. We only included articles in English due to resource limitations. There was no limit on the date of publication with the search carried out up to November 22, 2023. We included both empirical sources as well as government and organizational sources (e.g., health system reports) that included proposed, developed, or currently used planetary health-related frameworks within health-systems settings (see Table 2). We defined frameworks for the purpose of this review as any framework or model that proposes a structure around which planetary health within health systems can be considered or organized. Planetary health is “a field focused on characterizing the linkages between human-caused disruptions of Earth’s natural systems [e.g., climate change, biodiversity loss, pollution] and the resulting impacts on public health” [14]. Planetary health for this review was used as an umbrella term for anything specific to the health of the planet inclusive of climate change and environmental sustainability. With this, planetary health did not need to be explicitly named within the framework, however, frameworks had to focus on some aspect of planetary health within health systems (e.g., climate change, biodiversity, etc.). Given the potential dearth of available frameworks, we felt it was important to be more inclusive given the nature and purpose of the scoping review (i.e., what frameworks are currently available?). We did not include frameworks that were solely focused on planetary health education of health professionals. We did, however, include any articles in which planetary health education of health professionals was a part of an overarching planetary health-related health system framework. We also applied this to any health system frameworks that had some level of focus outside of planetary health-related elements (e.g., social, economic components). In these cases, we did include frameworks that were not only specific to planetary health, yet we only focused on the elements relevant to planetary health for the analysis.

**Table 2:** Summary of the inclusion criteria for the scoping review.

	Inclusion criteria
*Source type and date*	English language; empirical sources as well as government and organizational sources (e.g., health system reports) that included proposed, developed, or currently used planetary health-related frameworks within health-systems settings. No limit for publication date.
*Planetary health focus*	Planetary health as a concept for this review was used as an ‘umbrella term’ for anything specific or related to planetary health itself (e.g., climate change, biodiversity, and environmental sustainability).
*Frameworks*	Any framework or model that proposed a structure around which planetary health-related elements within health systems could be considered or organized. Frameworks that were not only specific to planetary health were included, yet only if they also focused on elements relevant to planetary health (e.g., climate change, biodiversity, environmental sustainability). Frameworks that were solely focused on planetary health education were not included unless planetary health education was a part of an overarching health system framework.

We engaged a two-stage article review process with the first title and abstract screening stage having 100% double screening by two independent reviewers (NR, KW). Any discrepancies were resolved by discussion with a third reviewer (STC or JHB). One reviewer (NR) completed the full text screening stage, and a second reviewer (KW) was brought in for audit purposes for 10% of the articles, with any discrepancies resolved by discussion with a third reviewer (STC or JHB).

### Data characterization, synthesis, and summary

Data extraction was carried out by one reviewer (NR) with a second reviewer cross-checking a random sample of 10% of the articles (KW). All article charting was done in Excel 365, and included: general article information, geographic location, level/sector of health system, framework name, framework description, framework purpose (e.g., explanatory, interactive, action-orientated [i.e., focusing on the decision or policy-making process]), stage of implementation (e.g., conceptual or operational), and accountability metrics when described (e.g., specific indicators or measures to keep track of progress towards planetary health-related goals). As the review aimed to obtain a broad overview of the work in this area, including empirical and grey literature, quality assessment of the included articles was not undertaken and all relevant work was reported. The frameworks reported in the included articles were then analyzed by one reviewer (NR) using content analysis [15] to identify the different domains, with a second author consulted for regular coding audits (STC or JHB). With this, we first carried out open coding of the included article frameworks, then, we organized the data with the purpose of reducing the initially identified categories (i.e., sub-categories) into higher-order categories representing the data. In accordance with the Arksey and O’Malley (2005) [10] scoping review methodology, we have provided a narrative account of findings organized thematically.

## Results

**Fig 1:**
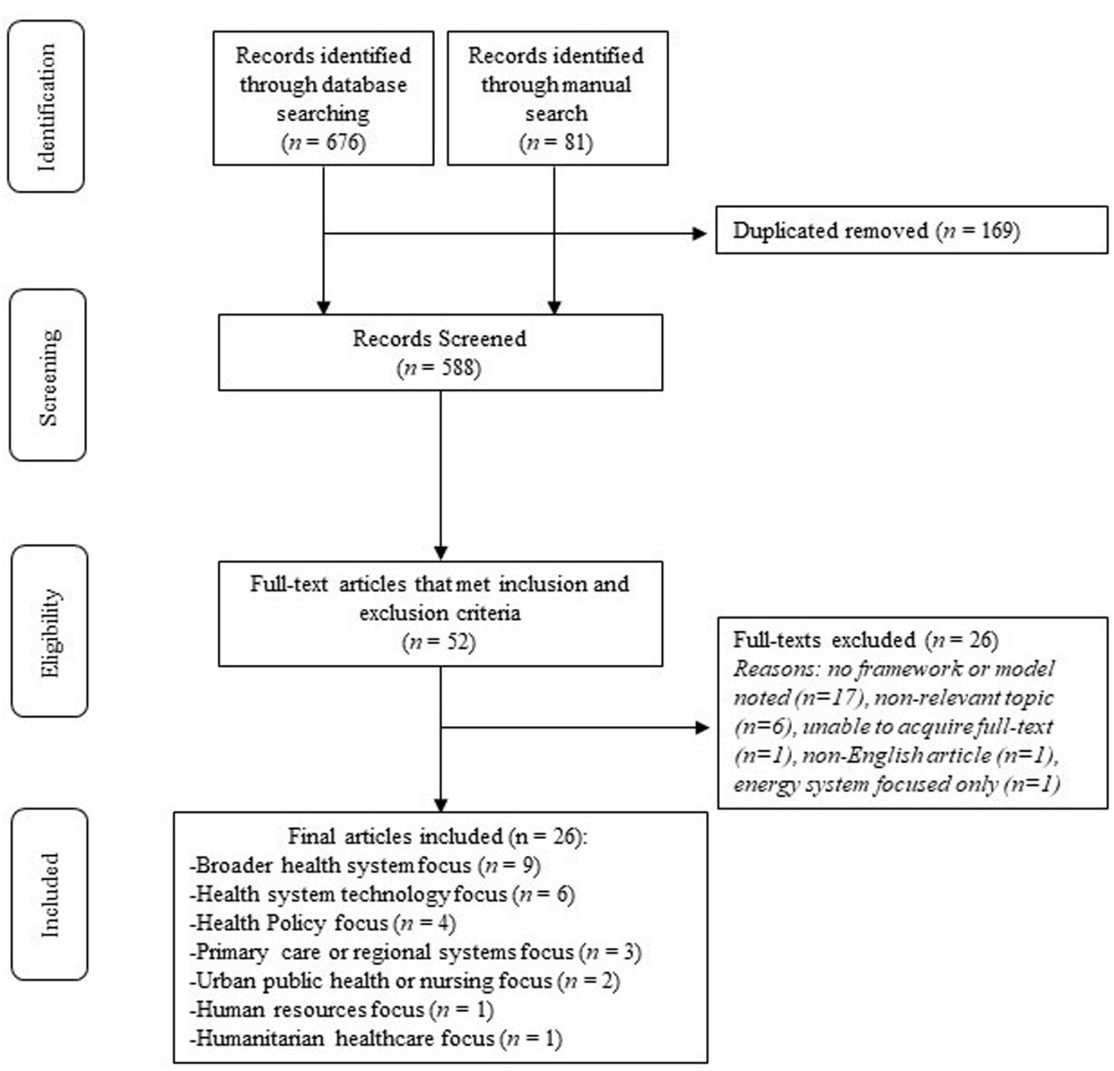
Adapted PRISMA diagram.

The systematic search identified 26 articles that met our inclusion criteria (see Fig 1). Most of the articles had a general health system focus (*n* = 9), or a health system technology focus (*n* = 6) (see Fig 1), with only ten articles specifying a geographic region of focus (e.g., India [16,17], Canada [18], Netherlands [19], Palestine [20], Australia [21], Thailand [22], United States [23], Romania Moldovan [24], Federated States of Micronesia/Marshall Islands/Palau [25]).

All but three articles were published in the last ten years, with most published around 2020. Most of the frameworks were conceptual in nature (*n* = 22), with only two noting explicitly that they had been validated [16,17]. Other included frameworks had been partially “operationalized” [18,22] or had been built or adapted from an existing framework that was most often used outside the context of planetary health [25–28]. Only seven of the included articles noted some level of measurement indicator(s) for assessing progress or accountability from implementation [7,20–22,24,29,30]. All the frameworks were developed to be action-orientated with only one stating explicitly that their development was theory-based [31]. A few of the frameworks additionally contained broader sustainability elements (e.g., economic sustainability, social sustainability) in addition to environmental sustainability components [16,17]. There was also variability in the terminology used within the names of the included frameworks with some ‘explicitly’ including the term planetary health, and others using proxy terms such as sustainability or green within the titles or framing. See Table 3 for key article characteristics and Table 4 for the included frameworks’ main health system focus areas mapped to the respective included article (see S1 Table for full data extraction sheet).

**Table 3:** Characteristics of included articles.

	Year	Level of health system^*^	Framework name	Framework description
*Bell* [32]	2011	Health policy level	A whole-of-systems approach to developing health services for climate change	The whole-of-systems approach describes health system responses in terms that involve a wide range of health and community services that could feasibly be part of whole of- government or even whole-of-community responses to climate change. This approach includes strategic, political, and economic responses, not just narrow health interventions.
*Chalabi & Kovats* [29]	2014	Health policy level	Health Adaptation Framework	A new generic conceptual framework was developed for development-compatible climate policy planning to evaluate policy options for middle- and low-income countries that reduce the adverse health effects of climate change. The proposed framework, incorporating system dynamics, provides a foundation for a decision-analytical approach to support the formulation of robust climate change adaptation policies to protect human health.
*Cimprich et al* [33]	2019	General health system level	Industrial ecology framework for healthcare sustainability	An industrial ecology framework was developed for healthcare sustainability. The framework conceptualizes the healthcare sector as comprising “foreground systems” of healthcare service delivery that are dependent on “background product systems.”
*Desmond* [34]	2016	General health system level	Understanding climate change mitigation efforts: a conceptual model	A conceptual framework is proposed that offers a model for the pursuit of sustainable development practice in health services. A set of propositions is advanced to provide a systems approach to assist decision-making by decoding the challenges faced in implementing sustainable health services.
*Dhaini et al* [35]	2023	Health system technology level	Intelligent green e-healthcare model/Green virtual machines (VM) Placement Model	A proposed segmentation model and an energy aware virtual machines (VM) placement methodology for an Intelligent and Green E-healthcare Model for an Early Diagnosis of Medical Images as an Internet of Medical Things (IoMT) is described. The proposed models are essential parts of an intelligent green e-healthcare model for an early diagnosis of Medical images as an IoMT application.
*Falceto de Barros et al* [26]	2022	Primary care level	The patient-centered planetary health (PH) care framework	A conceptual framework of patient-centered planetary health (PH) care is presented as essential for PH action at the community level. It is a reflective analysis of a clinical case report and describes and analyzes a clinical case of patient-centered PH care through 8 lenses.
*Gupta et al* [16]	2023	General health system level; India	Analytical Hierarchy Process (AHP) Model of Sustainable Healthcare	The research identified the factors associated with sustainable healthcare systems through a literature review, then a questionnaire was developed and administered to 25 healthcare organizations. An Analytical Hierarchy Process was utilized to rank the factors and a conceptual model was developed.
*Hussain & Subramoniam* [36]	2012	Health system technology level	Conceptual Model	A model is proposed for implementing ‘Information and communication technology’ (ICT) techniques in the healthcare industry to make the hospitals go green. Healthcare practitioners can use this model in understanding issues related to attaining competitive advantage via ICT. Researchers can use this model as a rough sketch to further explore the relationships between development in ICT and green healthcare.
*Kaladharan et al* [17]	2023	Health system technology level; India	Sustainability triangle framework for digital health systems	A conceptual framework is proposed called the sustainability triangle framework for digital health systems (DHS) which includes an environmental component. At the core, it shows a sustainable digital health system connecting the three forms of sustainability.
*MacNeill et al* [37]	2021	General health system level	Planetary healthcare: a framework for sustainable health systems	A framework for constructing environmentally sustainable health systems is presented. Three principle elements of the framework are: reduce demand for health services; match supply of health services to demand; reduce emissions from supply of health services.
*McIver et al* [25]	2017	National health system level; Federated States of Micronesia, Marshall Islands, Palau	Healthy Islands framework for climate change adaptation	The framework draws upon real-world experience and governance theory from both the health and climate change literature and, for the first time, places health systems adaptation within the vision for ‘Healthy Islands’ in the Pacific region. The proposed mechanisms for this model are: cross-sectoral collaboration from multiple actors; inform policies; and enable the implementation of appropriate and effective adaptation and mitigation measures.
*Moldovan et al* [24]	2023	General health system level; Transylvania, Romania	H-S innovative framework	Domains of a new innovative framework were defined by collecting the latest medical practices related to environmental sustainability, designing indicators related to environmental responsibility and a matrix of indicators, followed by its validation in practice at an emergency hospital. The framework contains 57 indicators, of which 8 are dedicated to the environmental area.
*Mousa & Othman* [20]	2020	Human resource level; Palestine	Green Human Resource Management (GHRM) Framework	A framework was developed to provide policy makers with set guidelines on how to influence and implement green human resource management practices for maximised sustainable performance. The framework was constructed according to five stages: 1) sustainability policy; 2) planning for sustainability; 3) implementation; 4) evaluation; and 5) review and corrective actions.
*Northern Sydney Local Health District* [21]	nd	Regional health system level; Australia	NSLHD Planetary Health Framework	The framework is intended to provide direction for the monitoring, development, and implementation of planetary health related initiatives across the Northern Sydney Local Health District (NSLHD). The framework focuses on five priority domains: sustainable organisation; waste management and resource recovery; capital works and procurement; models of care; and people and places.
*Puntub et al* [22]	2023	Urban public health level; Khon Kaen City, Thailand	Framework for Climate-Resilient Urban Public Health Care Services	A novel framework is presented for operationalizing local public health care under the two future global mega challenges: urbanization and climate change. The framework assembles scenario planning and composite-indicator tools to fasten day-to-day public health operations and long-term strategic planning. The composite indicators provide a broader and deeper perspective of potential impact and promote spatial-network integration with other health determining realms that are more advanced than the existing practices.
*Rajagopalan et al* [27]	2023	General health system level	3-lens organizational framework	This framework includes three aspects: strategic (which includes processes and procedures); political (authority and power); and cultural (underlying attitudes and beliefs). It is stated that examining a sustainability agenda from the vantage point of the strategic, geopolitical, and cultural domains of HCOs, allows one to not only appreciate barriers that could impede progress but also shed light on pathways for successful transformation.
*Schenk* [23]	2019	Nursing level; United States	“WE ACT–PLEASE” Framework	A framework for environmental stewardship in nursing practice is introduced. The framework is positioned within a global context of planetary health and planetary-level environmental harm. The framework describes five content domains of pollution from healthcare sources: Waste; Energy/water; Agriculture/food; Chemicals; and Transportation (WE ACT). It identifies six key professional elements: Professional Obligation; Leadership; Education; Accountability; Science; and Engagement (PLEASE).
*Schwerdtle et al* [38]	2020	Humanitarian healthcare level	An Operational Framework for Building Climate-Resilient Humanitarian Health Systems	An adapted framework was developed aiming to increase the capacity of humanitarian organizations to protect health in an unstable climate. The World Health Organization (WHO) operational framework for climate-resilient health systems7 was adapted for a humanitarian health organization that delivers healthcare and additionally presented concrete case studies to demonstrate how the framework could be implemented.
*Sijm-Eeken et al* [31]	2022	Health system technology level	Green-MIssion (Medical Informatics Solutions) framework	A theory-based framework was developed to enhance and accelerate development, selection, and implementation of solutions mitigating the climate impact of healthcare organizations. Existing frameworks were combined to develop the Green-MIssion (Medical Informatics Solutions) framework. The framework classifies solutions into three categories: (1) monitor and measure environmental impact of a healthcare setting; (2) help create and increase awareness among employees and patients; and (3) interventions to reduce environmental impacts.
*Sijm-Eeken et al* [19]	2023	Health system technology level; Netherlands	SEIPS 2.0: a human factors framework (adapted to the implementation and adoption of Green MI solutions in hospitals)	Interviews were carried out with healthcare professionals from Dutch hospitals to gain insights into which organizational and human factors impact the implementation and adoption of sustainable solutions. Key factors mentioned are formalizing tasks, allocating budget and time, creating awareness and changing protocols to promote sustainable diagnosis and treatment procedures. These elements were transcribed onto the SEIPS 2.0 quality improvement model.
*Sittig et al* [29]	2022	Health system technology level	Information technology-enabled Clinical cLimate InforMAtics acTions for the Environment” (i-CLIMATE)	A framework is introduced to illustrate how clinical informatics can help reduce healthcare’s environmental pollution and climate-related impacts. Actionable components of the framework include: (1) create a circular economy for health IT; (2) reduce energy consumption through smarter use of health IT; (3) support more environmentally friendly decision making by clinicians and health administrators; (4) mobilize healthcare workforce environmental stewardship through informatics; and (5) Inform policies and regulations for change.
*Sood & Teherani* [28]	2022	General health system level	Expanded sustainability framework for health system science (HSS)	An expanded sustainability framework for health systems science (HSS) is put forward to promote health systems’ capacity to deliver efficient, effective care for patients and to care for the planet by decreasing emissions and solid waste while cutting costs. Alignment of an expanded HSS framework that accounts for waste from medical outputs with existing HSS infrastructure (e.g., medical education curricular design) is stated to be achieved by applying Lean Six Sigma (LSS).
*Strashok et al* [18]	2010	National health system level; Canada	Green Policy Framework-Decision-Making Continuum	This paper provides an overview of the Canadian medical community’s recommendations for a green policy framework. The green policy areas discussed are a compilation of visions and strategies from many of the organizations in the sector, as well as key documents.
*World Health Organization* [30]	2020	General health system level	Framework for building climate-resilient and environmentally sustainable health care facilities	The proposed framework aims to increase the climate resilience of health care facilities to protect and improve the health of their communities in an unstable and changing climate, while optimizing the use of resources and minimizing the release of wastes by becoming environmentally sustainable. There are three objectives under each of the four fundamental requirements to provide safe and quality care that are central to the action framework.
*World Health Organization* [7]	2015	General health system level	WHO operational framework for building climate resilient health systems	The framework responds to the demand from Member States and partners for guidance on how the health sector and its operational basis in health systems can systematically and effectively address the challenges increasingly presented by climate variability and change. The objective of this framework is to provide guidance for health systems and public health programming to increase their capacity for protecting health in an unstable and changing climate.
*Xie et al* [40]	2018	Primary care level	Framework for primary care actions to create health co-benefits, and mitigate or adapt to the health effects of climate change	The developed framework provides clear parallels between changes in health behaviours (e.g., smoking, exercise, nutrition) and climate change adaptation and mitigation strategies. The framework outlines how primary care providers (PCP) can serve the community as strong promoters of actions that encourage environmental change.

* Where applicable the country context is noted.

**Table 4:** The frameworks’ health system main focus area within the included articles.

Framework Focus Areas	Relevant Articles
General health system focus	*Cimprich et al* [33], *Desmond* [34], *Gupta et al* [16], *MacNeill et al* [37], *Moldovan et al* [24], *Rajagopalan et al* [27], *Sood & Teherani* [28], *World Health Organizatio*n (2015) [7], *World Health Organization* (2020) [30]
Health system technology focus	*Dhaini et al* [35], *Hussain & Subramoniam* [36], *Kaladharan et al* [17], *Sijm-Eeken et al* (2022) [31], *Sijm-Eeken et al* (2023) [19], *Sittig et al* [39]
Health policy focus	*Bell* [32], *Chalabi & Kovats* [29]
Primary care focus	*Falceto de Barros et al* [26], *Xie et al* [40]
National or regional health system focus	*McIver et al* [25], *Northern Sydney Local Health District* [21], *Strashok et al* [18]
Urban public health focus	*Puntub et al* [22]
Nursing focus	*Schenk* [23]
Human resources focus	*Mousa & Othman* [20]
Humanitarian healthcare focus	*Schwerdtle et al* [38]

Content analysis of the included articles identified six overarching categories, and eleven sub-categories within the planetary health-related frameworks reviewed (see Tables 5 and 6). We review these in further detail below.

**Table 5:** Overview of the categories and sub-categories identified within the included planetary health-related frameworks.

Categories	Sub-categories
*1. The bi-directional impacts between health systems and the environment*	N/A
*2. Vision, advocacy, leadership, and communication for environmentally sustainable health systems*	2.1 Reasons for making planetary health-informed changes within health systems
	2.2 Culture, attitudes, and beliefs aligned with environmentally sustainable health systems
	2.3 Focus on co-benefits for human and environmental health
*3. Key structural components for environmentally sustainable health systems*	3.1 Economic elements
	3.2 Social elements
	3.3 Political, health policy, and governance elements
	3.4 Models of care
*4. Climate resiliency and environmental sustainability of healthcare facilities and systems*	4.1 Green performance management, environmental sustainability planning and implementation practices
	4.2 Health workforce mobilization
	4.3 Emergency preparedness and management
	4.4 Recycling, pollution control, and waste management programs with the stewardship of resources and sustainable consumption
*5. Climate-resilient and sustainable technologies and digital infrastructure*	N/A
*6. Evaluation and accountability within environmentally sustainable health systems*	N/A

**Table 6:** The categories mapped to the included articles.

Categories	Relevant Articles
*1. The bi-directional impacts between health systems and the environment*	*Chalabi & Kovats* [29], *Cimprich et al* [33], *Hussain & Subramoniam* [36], *Schenk* [23], *Sijm-Eeken et al* (2022) [31], *Sijm-Eeken et al* (2023) [19], *Sood & Teherani* [28], *Strashok et al* [18], *Moldovan et al* [24], *World Health Organization* (2020) [30], *Xie et al* [40]
*2. Vision, advocacy, leadership, and communication for environmentally sustainable health systems*	*Bell*, *Chalabi & Kovats* [29], *Desmond* [34], *Falceto de Barros et al* [26], *McIver et al* [25], *Moldovan et al* [24], *Rajagopalan et al* [27], *Schenk* [23], *Schwerdtle et al* [38], *Sijm-Eeken et al* (2022) [31], *Sijm-Eeken et al* (2023) [19], *Strashok et al* [18], *World Health Organization* (2015) [7], *World Health Organization* (2020) [30], *Xie et al* [40]
*3. Key structural components for environmentally sustainable health systems*	*Bell*, *Chalabi & Kovats* [29], *Cimprich et al* [33], *Desmond* [34], *Falceto de Barros et al* [26], *Gupta et al* [16], *Hussain & Subramoniam* [36], *Kaladharan et al* [17], *MacNeill et al* [37], *McIver et al* [25], *Moldovan et al* [24], *Mousa & Othman* [20], *Northern Sydney Local Health District* [21], *Puntub et al* [22], *Rajagopalan et al* [27], *Schwerdtle et al* [38], *Sijm-Eeken et al* (2022) [31], *Sijm-Eeken et al* (2023) [19], *Sittig et al* [39], *Sood & Teherani* [28], *Strashok et al* [18], *World Health Organization (2015)* [7], *Xie et al* [40]
*4. Climate resiliency and environmental sustainability of healthcare facilities and systems*	*Bell*, *Chalabi & Kovats* [29], *Cimprich et al* [33], *Desmond* [34], *Gupta et al* [16], *Kaladharan et al* [17], *MacNeill et al* [37], *McIver et al* [25], *Moldovan et al* [24], *Mousa & Othman* [20], *Northern Sydney Local Health District* [21], *Puntub et al* [22], *Rajagopalan et al* [27], *Schenk* [23], *Schwerdtle et al* [38], *Sijm-Eeken et al* (2022) [31], *Sijm-Eeken et al* (2023) [19], *Sittig et al* [39], *Sood & Teherani* [28], *Strashok et al* [18]*, World Health Organization* (2015) [7], *World Health Organization* (2020) [30], *Xie et al* [40]
*5. Climate-resilient and sustainable technologies and digital infrastructure*	*Dhaini et al* [35], *Hussain & Subramoniam* [36], *Kaladharan et al* [17], *MacNeill et al* [37], *Sijm-Eeken et al* (2022) [31], *Sijm-Eeken et al* (2023) [19], *Sittig et al* [39], *Sood & Teherani* [28], *World Health Organization* (2015) [7]
*6. Evaluation and accountability within environmentally sustainable health systems*	*Gupta et al* [16], *Hussain & Subramoniam* [36], *Kaladharan et al* [17], *Moldovan et al* [24], *Mousa & Othman* [20], *Puntub et al* [22], *Schenk* [23], *Schwerdtle et al* [38], *Sijm-Eeken et al* (2023) [19], *Sittig et al* [39], *Sood & Teherani* [28], *World Health Organization* (2015) [7], *World Health Organization* (2020) [30], *Xie et al* [40]

### 1. The bi-directional impacts between health systems and the environment.

Fewer than half of the identified frameworks explicitly included elements acknowledging the connection and the bidirectional impacts between health systems and the environment. This was both from the standpoint of highlighting key elements of how health systems affect the environment as well as how global environmental change can affect health systems. Climate change was most often highlighted with “inside-out impacts” (i.e., health system impacts on the environment), and “outside-in impacts” [33] (i.e., climate change impacts on health systems) most often noted. Examples of “inside-out impacts” included greenhouse gases and air pollution, impacts on water and sanitation, chemical and other waste production, resource depletion [28,30,31,33]—with only one framework explicitly identifying biodiversity effects [23]. Examples of “outside-in impacts” included floods, fires, heatwaves, temperature extremes, heat-related illness, sea-level rise, drought, storms, raw material supply disruptions, and climate-sensitive disease outbreaks [30,31,33,40]; with some frameworks noting just the generic element of “climate change” [23] or the “environment” [18,24,36].

### 2. Vision, advocacy, leadership, and communication for environmentally sustainable health systems.

Just over half of the included frameworks had explicit elements noted around vision, advocacy, leadership, communication for environmentally sustainable health systems either as isolated components or in combination. Participation and inclusiveness was platformed as being important [22] for progress forward with different levels of leadership highlighted as being necessary, from health providers to community leadership [23,39]. This leadership was contingent and connected to having health system supports in place for clinician and administrator decision-making around environmental sustainability. The health system itself was also highlighted as needing to lead by example by having a strategic profile that ensures climate-aware leadership approaches are in place across the system [34]. This could include, as one example, having recognized green teams and/or individuals across the health system [19]. Ultimately, it was noted that health systems needed to have a clear vision [19] and focus [16–18,23,29–31] on environmental sustainability, with environmentally sustainable healthcare being the default. To better ensure success, it was highlighted that there should be formalized communication structures in place across the health system [19,30], while ensuring appropriate engagement on the topic [23]. Three additional sub-categories were highlighted across the framework elements of vision, advocacy, leadership, and communication, which are discussed briefly below.

**2.1. *Reasons for making planetary health-informed changes within health systems:*** Nine of the included frameworks included specific reasons for making planetary health-informed changes within health systems. These included such things as: health professional obligation or intrinsic motivation [19,23,38], to reduce poverty incidence and inequities [29,40]; to preserve cultural heritage [29]; and to alleviate suffering, save lives, and reduce impacts on vulnerable populations [38]. It was also highlighted that health systems have a responsibility for patients impacted by climate change [31,34,40], and that they should be held to higher orders including doing things for the greater good [18]. It was noted that health systems are supposed to have a humanitarian focus—ensuring the platforming of human rights and maintaining human dignity by ensuring a healthy environment [24,38] through environmental protection and responsibility (i.e., protecting biodiversity, water, environmental resources) [23,24,29,40].

**2.2. *Culture, attitudes, and beliefs aligned with environmentally sustainable health systems:*** Within the included frameworks, the culture of health services was stated to need to “adapt to meet the demands of climate change” [32]. This cultural adaptation towards environmentally sustainable health systems included attending to the underlying attitudes and beliefs that exist within health systems [27], while also considering the different mechanisms for change processes that are inclusive of behavioral elements [32]. Frameworks noted that to promote a “green culture” [19] there needed to be a clear awareness of the specific actors involved in and around health systems who were often explicitly named within the included frameworks. Specific actors highlighted in addition to health system actors themselves were civil society organizations, communities, NGOs, other government agencies, and regional and international agencies [24,25]. The importance of cultural competency within primary care [26] was additionally called out as being an important attribute for planetary health-informed health systems. Lastly, when positive health systems change was occurring, it was highlighted that sharing successes and lessons learned was important to further facilitate positive environmental change [19].

**2.3. *Focus on co-benefits for human and environmental health:*** One framework stated the importance of understanding that healthy people are only possible within a healthy environment [30]. The ability to have co-benefit approaches through having climate-informed health programs [7,38] within health systems was additionally platformed as being critical for environmentally sustainable healthcare. Interestingly, a few frameworks specifically called out agricultural elements, including food systems [23,40], as also being a critical component of consideration for environmentally sustainable health systems. From a co-benefit approach this was from the standpoint of understanding that having a healthy environment means healthy and more nutritious foods for people within and outside health systems.

### 3. Key structural components for environmentally sustainable health systems.

Most of the included articles in the review noted or platformed varied structural components critical to ensuring environmentally sustainable health systems. These elements are reviewed below.

**3.1. *Economic elements:*** Several frameworks had clear economic components including: 1) matching supply to demand of health services while redefining value to ensure environmental sustainability is considered [37]; making the business case with environmental sustainability as a business strategy [27]; the need for circular economy in supply chains, and climate-smart supply and procurement systems [21,37–39]; reengineering the business processes surrounding health systems towards environment sustainability [36]; ensuring climate and health financing with improved economic performance and fiscal sustainability [7,28,32]; ensuring there are asset management skills in place for anticipated climate impacts [32]; the need to platform domestic production for climate resiliency within health systems [16]; and finally, the potential for increased employment and productivity as well as the creation of “additional jobs in the health sector for developing and implementing the adaptation and mitigation options” within an environment of climate change [29].

**3.2. *Social elements:*** Some of the included frameworks highlighted the social determinants of health as being an important element for environmentally sustainable health systems [7,28]. Much of this stemmed from the understanding that the social determinants of health affect “the vulnerability of populations to climate change and their capacity to adapt” [40]. The need for a social determinants lens within health system management in the context of climate change was seen to be an inherent social responsibility [24,34]. This determinant of health approach also made space for environmental sustainability processes to be focused on people and places, including being family orientated [21,26]. Interestingly, across the frameworks there was minimal explicit mention of community engagement for environmentally sustainability health systems; however, there was note of stakeholder consultations and community surveys being carried out to support climate-related project work [25]. One framework noted the importance of social capital for bonding and bridging climate change and health-related networks [25]. Another noted the importance of digital social networking for professional interaction on the topic [36].

**3.3. *Political, health policy, and governance elements:*** Several included frameworks included elements reflective of political, health policy, and governance landscapes. This included ensuring that the regulatory and financial environment, legal requirements, and formal legislation (laws) are conducive to promoting and better ensuring the context and backing is set for enabling environmentally sustainable health systems [19,25,33,34,36,39]. There was specific note on the importance of having a supportive external policy environment that included national governments platforming sustainable healthcare [19], optimal insurance rules [31] and hospital accreditation standards for environmental sustainability [24], as well as the explicit need for political stability, appropriate authority, and enabling power for change at the political level [27,29,34,36].

Policy implementation considerations and needs were highlighted as being important for environmentally sustainable health systems. There was a stated need to create structures to allow for easy policy implementation while complying with the required optimal timing of policy interventions for best results [29]. This could be enabled through formalized tasks within the system for green actions led by green teams [19], and by being aware of interdisciplinary rivalries and power differentials that could prevent needed action [34]. At the governance level, there was a stated need for climate-aware organizational governance [7,21,31,34,38] with effective decision-makers in place to enable change [20]. With climate-aware organizational governance, climate and sustainability-specific policies and procedures [20,28] could be enacted with a preference for a “whole-of-systems policy response” [32] with strategic development plans [25] in place to enable environmentally informed change.

**3.4. *Models of care:*** Within the umbrella of environmentally sustainable health systems, effective models of care were highlighted as being important within the included frameworks. Alongside the need for environmental considerations, the what, where, and how care is delivered still matters (e.g., fair healthcare practices) [7,24,32–34]. This included ensuring access, continuity, coordination, and comprehensive patient-centered care [26,37]. There was clear understanding that when you can improve human health (e.g., health promotion), it can reduce the overall demand on carbon and environmentally intensive care [25–37]. Health promotion and health prevention activities to reduce healthcare needs were partly enabled by ensuring well-supported primary care-orientated models of service delivery [37,40]. Additional leveraging of value-based care was seen to reduce defects in system processes by better controlling for variation in carbon-intensive healthcare delivery [24,28]. With supporting management functions and activities in place to influence clinical decisions towards environmentally sustainable healthcare (e.g., procurement, waste management, administrative services, food service) [33], planetary or green healthcare and implementation practices would be more easily enabled [18,26]. Lastly, moving towards models of care that uplift regenerative and restorative principles was noted as being essential, with the ongoing understanding that it is an environmental imperative to live within the global biophysical carrying capacity [18].

### 4. Climate resiliency and environmental sustainability of healthcare facilities and systems.

Included frameworks highlighted aspects of embedding climate resiliency and environmental sustainability into both healthcare facilities and systems. The need for overall climate-related adaptation of infrastructure, processes, technologies, and products were repeatedly highlighted. This could be regarding the promotion of new systems and technologies for climate-related adaptation and mitigation, or specific management and system processes to facilitate the movement towards environmental sustainability of healthcare operations and facilities [30,37]. Below, we highlight key sub-categories that were represented within the included frameworks for this section.

**4.1. *Green performance management, environmental sustainability planning and implementation practices:*** Close to half of the frameworks included management, planning, and implementation practices in relation to environmentally sustainable health service delivery. For environmental sustainability planning and implementation to occur, there was a noted need for movement towards innovation and systems change backed by strategic transformation processes [27]. These change processes could be leveraged by utilizing established approaches such as systems change approaches and Lean Six Sigma methodologies [28]. Additional management schemes that were noted to help support the context for climate resiliency within planning and implementation included systems that had: built in flexibility and modularity; were responsive; supported policies around appropriate integration and coordination of service delivery; and that mainstreamed climate-risk within the systems themselves [22].

**4.2. *Health workforce mobilization:*** The ability to build coping and adaptive capacity in terms of climate-related events and health system resiliency was dependent on the health workforce being able to be mobilized to respond [22]. Green human resources practices were found to have had a positive influence on environmentally sustainable performance [20]. This was through on-the-ground operational practices such as having specific environmental goals adopted by every manager and employee in the organization, rewarding staff when they improve environmental programs, or rewarding staff when they come up with “remarkable ideas” [20]. Having green hiring in place across the health system was an additional element highlighted. Specific green hiring practices included having job descriptions include environmental aspects, job interviews including the testing of knowledge on the environment, and specifically recruiting employees who have knowledge about the environment [20]. Three frameworks included the need for green training, education, and empowerment within the existing and future health workforce [23,30,40]. Having a climate-informed workforce and workforce development process in place [32,38] (e.g., carbon literacy and systems support [34]) would better ensure the overall familiarity with green practices and capacity development across the system [30].

**4.3. *Emergency preparedness and management:*** Some of the included frameworks made specific reference to climate-related emergency preparedness and management as key facets for building climate resiliency and environmental sustainability within healthcare systems and facilities. For effective emergency preparedness and management in this regard, the need to mainstream climate-risk in planning processes across the system was highlighted [22]. Vulnerability, capacity, and adaptation assessments were specifically flagged in this sub-category as being a critical piece for health systems to prioritize [7]. In addition, prioritizing integrated risk monitoring and management, as well as early warning and response within the health system was noted [7,30,38].

**4.4. *Recycling, pollution control, and waste management programs with stewardship of resources and sustainable consumption:*** Some frameworks highlighted processes to reduce the overall impact of healthcare facilities and systems on the planet. This was most often regarding the need to reduce energy consumption and emissions (e.g., decarbonized energy and transportation systems), as well as reducing pollution and waste (e.g., supporting systemic change towards a circular economy [i.e., “a model of production and consumption, which involves sharing, leasing, reusing, repairing, refurbishing and recycling existing materials and products as long as possible” [41]) from health services [30,33,37,39,40]. There was a noted need to be strategic in how healthcare resources were mobilized while being responsive to health system needs and reducing redundancy in the overall system for better efficiency [22]. This was supported by, for example, enabling the reprocessing of used medical devices and resource recovery processes that enabled more sustainable consumption [21,33]. Other examples were ensuring cleaner overall production and disposal methods within procurement, supply chains, and waste management stages [17,30,37]. Overall, however, it was highlighted that minimizing overall waste and resource use (e.g., water) while limiting environmental costs and adding value to patients within the health system was needed (i.e., resource stewardship and environmentally preferable practice) [28].

### 5. Climate-resilient and sustainable technologies and digital infrastructure.

Several of the articles were focused on the climate resiliency and overall environmental sustainability of the technologies and digital infrastructure used within health systems. This was both from the standpoint of how technologies and digital infrastructure could help to reduce healthcare’s environmental impact (e.g., improving efficiency) [7], but also by being mindful of the high energy usage needed for many technologies as well as the production of electronic waste (i.e., e-waste), and how to better mitigate this [39]. In moving towards climate-resilient and sustainable technologies and infrastructure, several operational examples were provided that were dependent on health system awareness and knowledge of IT solutions including: the use of integrated technology systems (e.g., medical devices connected to information technology [IT]) [35,37]; making use of green dashboard and footprint data for monitoring and improvement work [19]; leveraging expert systems and knowledge platforms to yield reductions in errors and improve efficiencies [19,36]; and the use of cloud infrastructure with fog computing (i.e., a form of distributed computing) to support medical alerts, notifications, and monitoring to improve early diagnostics in a green-aware fashion [35].

Frameworks also highlighted the important application of technology interventions for environmental sustainability within health systems. These included telehealth (virtual care) and the efficient use of electronic health records (EHR)/electronic medical records (EMR), as well as having more effective data-sharing processes in place [19,36,37]. Other kinds of clinical informatics and technology were highlighted for consideration on the pathway towards better environmental sustainability including the internet of things (IoT) [35], picture archiving and communication systems (PACS), intelligent decision support systems (IDSS), artificial neural network, and clinical decision support systems (CDSS) [36]. Ultimately, there was need to be able to monitor and assess problems through IT architecture to support the movement towards environmentally sustainable health systems while being mindful of potential security challenges and the overall need for effective IT governance [19]. Appropriate implementation entailed balancing the potential efficiencies gained through less use of resources while designing systems that were also not resource and energy intensive themselves.

### 6. Evaluation and accountability within environmentally sustainable health systems.

Over half of the frameworks included elements connecting the importance of formal evaluation processes and accountability mechanisms alongside the movement towards environmentally sustainable health systems. Most commonly noted were the monitoring and evaluation of environmental performance and outcomes using a metrics-driven approach [17,22,28,30]. Environmental performance, however, was still seen to be connected to measuring overall hospital and patient outcomes, social performance, and patient and employee satisfaction alongside environmental outcomes [16,20,24,36]. Climate-informed needs assessments and sustainability scans, ongoing performance evaluations, and quality improvement processes were seen to be integral to expanding the work while ensuring effective progress [19,24,28,38]. Increased research and development was highlighted in several of the frameworks as being necessary to ensure evidenced-based environmental sustainability processes are grounded in appropriate evidence [7,16,38,40].

## Discussion

From the content analysis, we identified six overarching categories and eleven sub-categories in our framework review relevant to planetary health within health systems. These categories included: 1) the bi-directional impacts between health systems and the environment (e.g., emissions, medical waste, climate weather events); 2) vision, advocacy, leadership, and communication (e.g., climate-aware leadership and communication approaches); 3) key structural components for environmentally sustainable health systems (e.g., making the business case for environmental sustainability, inclusion of a social determinants of health lens, appropriate regulatory and financial environments in place) as well as models of care felt to be needed (e.g., value-based care, supported primary care systems); 4) climate resiliency and environmental sustainability of healthcare facilities and systems (e.g., climate-related adaptation of infrastructure, processes, technologies, and products); 5) climate-resilient and sustainable technologies and infrastructure (e.g., improving efficiency throughout the system); and 6) evaluation and accountability mechanisms (e.g., monitoring and evaluation using a metrics-driven approach).

Notable across the frameworks was that the vast majority were conceptual (*n* = 22), with only two being validated and two being only partially operationalized. It was therefore not surprising that only seven out of the twenty-six included frameworks explicitly included some level of measurement indicator(s) for assessing progress or accountability from an implementation lens. Most of the frameworks were also published quite recently with all but three articles published in the last ten years, with most published around 2020. Given this, we anticipate that more of the frameworks will be operationalized in varying contexts in the future. This would allow for a more detailed analysis of their uptake and impact. This would also give a better understanding of any potential challenges and barriers, or lessons learned that other health systems may learn from, as the planetary health field progresses within healthcare spaces. Concerning, however, was the dearth of explicit processes outlined within the frameworks or their respective descriptions for any community engagement carried out in the development of the frameworks themselves. Only three articles explicitly included recognition of some level of community orientation, involvement, or engagement within the frameworks [25,26,42]. A few others included some level of stakeholder engagement for their data-gathering process in development of the framework (e.g., survey) [22], or noted the importance of community engagement when considering local implementation processes for environmental sustainability within their descriptions [7,30]. Given the noted conceptual nature of many of the included frameworks, there appears to be substantial need for more community and stakeholder involvement in the development and refining of frameworks towards more informed implementation processes. Co-development and appropriate partnership processes should be engaged that platform local expertise and transdisciplinarity while involving patient and community facing stakeholders.

The included frameworks focused mostly on climate change, with pollution also being relatively well-represented. Biodiversity considerations were almost non-existent with only two mentioning the word ‘biodiversity’ in the context of either biodiversity loss or protection [23,29]. Frameworks were therefore almost completely devoid of concepts or domains related to nature and ecosystems, and the planet itself. With this, most of the frameworks only addressed some ‘components’ of planetary health without orienting fully towards planetary health more explicitly and comprehensively. The concept ‘green’ or ‘greening’ was the closest identification to nature within the frameworks [18,20,35,36], although it was often unclear how this framing was being interpreted in the context of nature. Given the increasing concerns around “greenwashing” within healthcare and other organizational and business spaces [43,44], clear and transparent reporting, as well as accountability mechanisms need to be put in place when considering planetary health-related implementation processes.

Additionally, wider environmental sustainability and climate change movements have been criticized for being overly mechanical and anthropocentric in nature (i.e., centering humans’ needs), and attempting to maintain the same standards of overconsumption that has perpetuated planetary harm [45,46]. Most of the frameworks included in our review appeared to focus on maintaining the status quo of health system operations, reinforcing a transactional approach to understanding health and health system function (i.e., human and techno-centric approaches). Therefore, there was a lack of interrogation of the cultural factors and values that underpin overconsumption and overuse of resources. A few frameworks did highlight the importance of investing in upstream drivers of health and well-being. This included the social determinants of health and ensuring adequate access to primary prevention and care [26,37,40], which decreases the need for complex care. All frameworks, however, were still framed or situated within Euro-Western value systems, epistemologies, and knowledge. Along these lines, none of the included frameworks explicitly stated the underpinning values or value systems from which they emerged. It has been argued that if one tries to tackle “an existential threat with the same epistemological underpinnings that caused the problem, the solutions will not create the change needed for the health of our biosphere” [45]. Thus, there is a strong argument for future planetary health-related frameworks to include nature and the planet more explicitly. Additionally, reflecting on the overall goals of environmental sustainability work and the value systems that underpin the work for deeper levels of change is needed. This does not, however, minimise, the need to utilise existing frameworks and their respective domains to work towards efficient and urgent change within health systems.

Overall, there were some comprehensive frameworks in our review that included many of the domains we identified [7]. Other frameworks were not as comprehensive yet went into more detail and depth on topic-specific areas that would be more helpful for department-level implementation than perhaps the more comprehensive ones (e.g., human resources, IT systems), reflecting differences in scope. From an operational standpoint, there was clearly more emphasis and focus on clinical care, with an acknowledged lack of research and information on important medical activities, supporting activities, background product systems, pharmacy services, hospitalization, ambulance services, and biomedical research in the context of environmental sustainability [33]. Given this, it becomes more difficult for health systems to prioritize change implementation processes (especially in resource-limited environments) outside of the larger more obvious planetary-health related needs (e.g., decarbonization, reduction in waste, etc.). We call upon regional, national and international governments, funding agencies, and organizations to support further research and implementation work around planetary health-informed health systems change while considering existing frameworks and the nuances inherent within regional contexts. We additionally call for increased attention towards health system frameworks that explicitly acknowledge planetary health more comprehensively (i.e., climate change, biodiversity loss, pollution), and not just ‘sustainability’ on its own.

To move this work forward, next steps should include the development of wise practices for incorporating other knowledge systems (e.g., Indigenous Peoples and their knowledges), as well as the development of clear pathways for ensuring the inclusion of other global environmental changes related to planetary health (e.g., biodiversity) into health system framework development. In addition, more concerted work is needed to identify the best levers for collaborating with community partners on this topic. We also see the value in carrying out more evaluation studies to better clarify the elements within the respective frameworks that are valuable for addressing the challenges of global environmental change. This work could ultimately lead to clearer, evidence-informed planetary health frameworks that are most effective and efficient in their transformation potential.

### Limitations

Given the varied use of terminology with the planetary health-related space broadly (e.g., sustainability, green, climate change, etc.), as well as our inclusion criteria being inclusive of English language articles only, there is a possibility that our search strategy missed relevant frameworks. Despite this, given this is the first review of this nature of which we are aware, with most of the included articles being published relatively recently, we feel we have captured some of the key categories for consideration in future work. Many of the identified categories were included across either different geographic domains or practice domains that helped ground them in varied contexts. Scoping reviews do have inherent limitations as their focus is to “provide breadth rather than depth of information in a particular topic” [47]. Given this, we focused on giving a high-level overview of the available framework components, as opposed to going into depth on selected specific domains. This means we may have inadvertently minimized some components of included frameworks. As our intention was not to create a hierarchy of different components of the frameworks (i.e., rate their importance or priority level), we feel our framework category summaries provide a preliminary overview of the key components to support future work. Finally, as this was a scoping review, we did not set out to critically appraise the framework development processes, instead focusing on providing a comprehensive overview of the frameworks available.

## Conclusion

Our scoping review identified several planetary health-related frameworks that may be useful to environmental sustainability activities within health systems, as well as domains that should be considered by organizations moving towards planetary health-informed structures and processes. Although we identified domains within our included frameworks that have more discussion and coverage around them in the context of health system environmental sustainability (e.g., carbon emission and medical waste reductions), we also identified domains that are generally less well-appreciated (e.g., green human resourcing, environmentally sustainable IT systems). We identified a greater need for community engagement processes in framework development as well as a need for more evaluation and accountability mechanisms built into environmental sustainability planning and practice (i.e., a metrics-driven approach). We also flagged the need for inclusion of *all* facets of planetary health (e.g., biodiversity), instead of solely focusing on climate change-related and pollution-informed health systems change. Lastly, further work is needed with other knowledge systems (e.g., Indigenous Peoples and their knowledge systems) to ensure planetary health-related frameworks are grounded in what we are trying to protect—the planet itself.

## Supporting information

S1 Table Full data extraction sheet. (XLSX)

S1 Checklist PRISMA checklist. (PDF)
